# Formation of amorphous silica nanoparticles and its impact on permeability of fractured granite in superhot geothermal environments

**DOI:** 10.1038/s41598-021-84744-2

**Published:** 2021-03-05

**Authors:** Noriaki Watanabe, Hikaru Abe, Atsushi Okamoto, Kengo Nakamura, Takeshi Komai

**Affiliations:** Department of Environmental Studies for Advanced Society, Graduate School of Environmental Studies, Tohoku University, Sendai, 9808579 Japan

**Keywords:** Geothermal energy, Hydrogeology

## Abstract

Superhot geothermal environments in granitic crusts of approximately 400–500 °C are a frontier of geothermal energy. In the development of such environments, there is a concern of a reduction of permeability of fractured granite due to the formation of fine particles of amorphous silica induced by the phase change from subcritical water to supercritical water or superheated steam. However, the formation of silica particles and a resultant reduction in permeability have not been demonstrated to date. Therefore, experiments were conducted on the formation of amorphous silica particles with various combinations of temperature (430–500 °C) and pressure (20–30 MPa), in which the phase change of Si-containing water from liquid to either supercritical fluid or vapor was induced. Amorphous silica nanoparticles occurred under all conditions with smaller particles for higher temperature. The permeability of fractured granite was also observed to decrease significantly within several hours during injection of the particles into rock at 450 °C and 30 MPa under a confining stress of 40 MPa, with slower permeability reduction at a smaller number of particles or in the presence of larger aperture fractures. The present study suggests that the nanoparticles are likely to form and destroy the permeability in superhot geothermal environments, against which countermeasures should be investigated.

## Introduction

Accessing geothermal environments at temperatures exceeding the critical temperature of water (pure water: > 374 °C, seawater: > 406 °C) at approximately 2–4 km depth is expected to increase productivity and sustainability in geothermal energy utilization because such superhot or supercritical geothermal environments, demonstrated by drilling in Italy^1–3^, Iceland^4–6^, the United States^7,8^, Mexico^9^ and Japan^10^, could provide supercritical water or superheated steam with high specific enthalpies of greater than or equal to ca. 2 MJ kg^−1^^11–16^. The superhot geothermal environments occur near the brittle–ductile transition zone in the continental granitic crust^17^. Significant aspects of the environments are the elevated efficiency of mineral plastic processes^18,19^, the retrograde quartz solubility^7,20,21^ and enhanced rates in the fracture healing and sealing by water–rock reactions^22–24^. These could cause the loss of the permeable fracture networks that transmit and store geothermal fluids. However, the networks are believed to form and last for a certain period^25–27^. Therefore, it is valuable to find enhanced geothermal system (EGS) technologies that are appropriate for superhot geothermal environments^28^ such as hydraulic fracturing to produce or reproduce permeable fracture networks through which a heat transmission fluid (i.e., water) circulates between injection and production wells, and means to keep the permeability of the fractured rock adequately high for profitable and sustainable energy production because permeability changes impact energy production in any type of geothermal system^29–33^.

Experiments on hydraulic fracturing of granite under triaxial stress, which involve the use of low-viscosity water at temperatures of ≥ 400 °C, have recently revealed the occurrence of a network of permeable fractures densely distributed throughout the entire rock volume^34,35^. Therefore, hydraulic fracturing has been confirmed as able to produce fracture patterns that would enable the effective utilization of superhot geothermal energy. There is, however, a concern about the viability of the high permeability of the fractured rock because a very recent study suggested that permeability may decrease as a result of the formation of fine amorphous silica particles when the phase change of water induces a highly supersaturated state of amorphous silica^36^.

Amagai et al.^36^ conducted so-called flash experiments, in which Si-containing water obtained by the dissolution of quartz and granite in water, situated at either the subcritical or supercritical state in an autoclave, was instantaneously discharged into the air and precipitates formed through the flash of water were caught by a filter. As a result, it was confirmed that amorphous silica occurred as nano- to microscale spherical particles by nucleation and aggregation during the evaporation of water. Moreover, the fine particles in hydrothermal solution rapidly changed to microcrystalline quartz via dissolution and precipitation. This change occurred within a day under supercritical conditions due to the large surface areas of fine particles. It was thus suggested that such silica particles can have a significant impact on the hydrology and mechanical behavior of hydrothermal systems in volcanic areas.

In the development of superhot geothermal environments, the phase change of water from subcritical water to either supercritical water or superheated steam inevitably occurs when water circulating between the injection and production wells as the temperature increase induces the phase change during the extraction of thermal energy. This implies the possibility of amorphous silica particle formation and transport of these particles through fractures in the rock, which leads to a reduction of rock permeability due to clogging of the fractures by the particles and their subsequent transformation into quartz. However, particles were previously obtained^36^ as a result of the uncontrolled rapid decrease of pressure and temperature by discharge of the high-temperature pressurized water into the air, which is far from the situation expected in superhot geothermal environments. Therefore, it is unclear if such nano- and microscale particles are actually formed in supercritical water or superheated steam in superhot geothermal environments. It is also unclear if such fine particles clog the fractures in rocks and reduce the permeability significantly, because no experiments on silica particle transport through high-temperature fractured rock have been conducted to date.

Therefore, the present study experimentally explores the formation of amorphous silica particles and their impact on the permeability of fractured granite under controlled temperature and pressure conditions that are considered to occur in superhot geothermal environments. Amorphous silica formation experiments were first conducted with various combinations of temperature (430, 450, and 500 °C) and pressure (20, 25, and 30 MPa), and amorphous silica transport experiments in fractured granite were then conducted at 450 °C and a pressure of 30 MPa under a confining stress of 40 MPa, for various combinations of initial rock permeability (approximately 1 × 10^–17^ and 1 × 10^–16^ m^2^) and Si concentrations in the injected water (approximately 200, 300, and 400 mg kg^−1^). We finally discuss the risk of permeability reduction due to formation of amorphous silica particles in the development of superhot geothermal environments, i.e., in superhot EGS.

## Experimental methods

### Si-containing water and granite sample

In both the amorphous silica formation and transport experiments, the formation of amorphous silica was attempted by achieving a supersaturated state of amorphous silica by the phase change of Si-containing water at room temperature to supercritical fluid or superheated steam. Si-containing water with a prescribed Si concentration was prepared from a Si-containing stock solution obtained from the dissolution of a mixture of quartz and granite sands in water at 360 °C and 35 MPa. The Si-containing stock solution contained Si at approximately 380 mg kg^−1^ and other elements at much lower concentrations, as summarized in Table 1. This solution was used either with or without dilution depending on the prescribed Si concentration in each experiment as described in “Amorphous silica formation experiment” and “Amorphous silica transport experiment” sections.

**Table 1:** Elemental concentrations of the Si-containing stock solution.

Element	Concentration (mg kg^−1^)
Al	2.7
Ca	1.0
Fe	< 0.1
K	2.5
Mg	< 0.1
Na	4.4
Si	380.6
Ti	< 0.1

**Figure 1:**
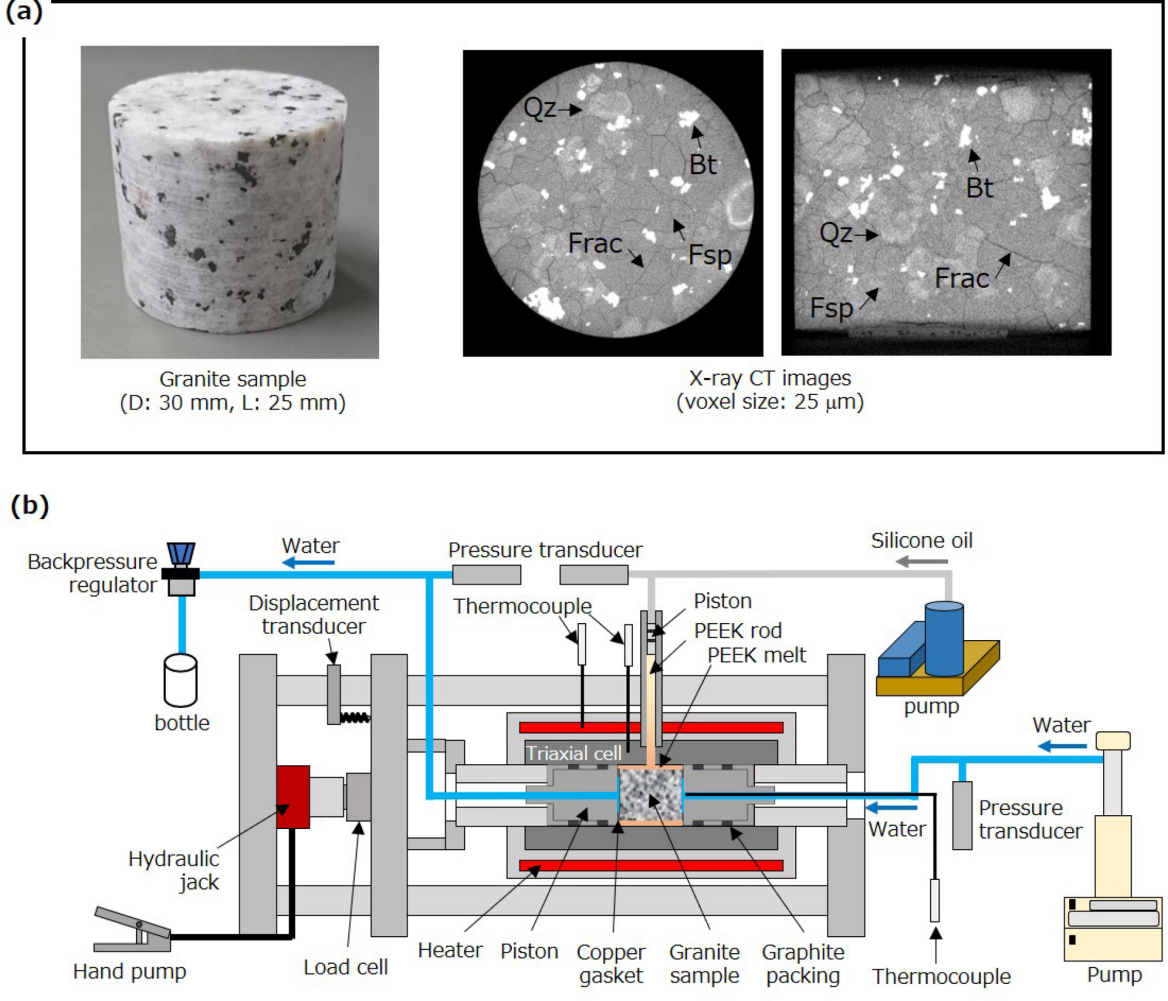
(**a**) Photograph and X-ray computed tomography (CT) images of the fractured granite sample and (**b**) schematic illustration of the system used for the amorphous silica formation and transport experiments. The CT images (Fsp: Feldspars, Qz: Quartz, Bt: Biotite, Frac: Fracture) were acquired at a X-ray tube voltage of 120 kV and a current of 150 μA using a micro-focus X-ray CT system (ScanXmate-D225RSS270, Comscantecno Co., Ltd.).

In the amorphous silica transport experiment, the Si-containing water was injected into a fractured granite sample. Cylindrical fractured granite samples (diameter: 30 mm, length: 25 mm) containing multiple fractures induced by heating in an electric furnace at 570 °C and atmospheric pressure for 2 h were prepared using Inada granite sourced from Ibaraki prefecture, Japan (Fig. 1a). The Young’s modulus, porosity, and permeability of Inada granite at or near atmospheric pressure are approximately 55–80 GPa, 0.5–0.8%, and 2–8 × 10^−18^ m^2^, respectively^26,34,35^. The modal composition of the major component minerals measured using a point counter was 36% quartz, 32% plagioclase, 28% alkali-feldspar, and 4% biotite^37^. The samples had some difference in permeability, which allowed investigation of the influence of initial permeability (i.e., number and/or aperture of fractures) on amorphous silica transport.

### Experimental system

An experimental system, developed by Watanabe et al.^26^ and used by Watanabe et al.^24,34^, was used for both the amorphous silica formation and transport experiments (Fig. 1b). The novelty of this system is the use of a special triaxial cell, which uses a high-viscosity plastic melt as a confining fluid and a thin plastic film as a sleeve. The plastic melt is composed of polyether ether ketone (PEEK), which has a melting point of 343 °C and a decomposition temperature greater than 538 °C. PEEK has a high viscosity of 350 Pa s, even at 400 °C, so that sealing the PEEK melt is easily achieved. The plastic film is polyimide (thickness: ca. 50 μm), which has no melting point (i.e., it decomposes before melting) and a high decomposition temperature of > 500 °C. The polyimide also acts as a release agent after the experiment. This triaxial cell can operate at approximately 350–500 °C when using PEEK and at lower temperatures with a plastic that has a lower melting point, such as polyethylene.

In the amorphous silica transport experiment, a fractured granite sample is wrapped in the polyimide film and first placed inside a PEEK cylinder within the triaxial cell. Copper gaskets with holes for fluid flow are attached at both ends of the sample. A tube containing a PEEK rod, which is used to inject the PEEK melt, is attached to the upper part of the cell. The cell is then placed within an electric furnace. A small axial pressure of ca. 2 MPa is maintained by a hydraulic jack, and the temperature is increased to a prescribed value, which melts both the PEEK cylinder and the rod inside the triaxial cell. Temperatures are measured both inside and outside the cell. A prescribed confining stress is then applied to the sample by adjustment of the confining pressure through the PEEK melt; the axial pressure is slightly higher than the confining pressure (the differential stress is approximately 2 MPa). The confining pressure is controlled by injecting the PEEK melt at a prescribed pressure through the upper tube using a metallic piston that is displaced by pumping silicone oil at a constant pressure. At the prescribed temperature and confining stress, water (pure water first, then Si-containing water) is injected into the sample at a prescribed flow rate using a pump. The water exits the sample at a prescribed water pressure controlled by a backpressure regulator, where the injected water is preheated to the prescribed temperature and becomes a supercritical fluid or superheated steam, depending on the backpressure before entering the sample. The sample permeability may be calculated using Darcy’s law based on the pressure, flow rate, and viscosity of water, and the cross-sectional area and length of the sample.

On the other hand, in the amorphous silica formation experiment, a cylindrical stainless-steel with a through-hole (3 mm in diameter and 40 mm in length) instead of the fractured granite sample was placed within the triaxial cell. No confining pressure was required in this experiment; therefore, the PEEK was not used. At the prescribed temperature, water (pure water first, then Si-containing water) is injected into the through-hole at a prescribed flow rate and a prescribed pressure with an appropriate axial pressure to activate the copper gaskets.

### Amorphous silica formation experiment

In the amorphous silica formation experiment, pure water was first injected at 0.3 mL min^−1^ at a prescribed temperature and pressure, and the injected fluid was then switched from pure water to Si-containing water with a prescribed Si concentration. The Si concentration was adjusted so that the supersaturation ratio of amorphous silica became 2 at that combination of temperature and pressure, by diluting the Si-containing stock solution with pure water. Note that the supersaturation ratio is the ratio of the Si concentration of Si-containing water to that based on the solubility of amorphous silica. The solubility of amorphous silica can be obtained at various combinations of temperature and pressure using predictive equations for the solubility ratio of amorphous silica to quartz in Karásek et al.^38^ and the solubility of quartz in Manning^39^.

Effluent was collected after pure water within the flow path was completely replaced by the Si-containing water. The effluent was filtered by a syringe filter (pore size: 0.22 μm) to collect solids in the effluent for observation and analysis using scanning electron microscopy (SEM; SU-8000, Hitachi, Ltd.) and energy dispersive X-ray spectroscopy (EDX; NORAN System 7, Thermo Fisher Scientific Inc.), while the filtered liquid was analyzed using inductively coupled plasma optical emission spectroscopy (ICP–OES; P-4000, Hitachi, Ltd.) for the elemental concentrations of Al, Ca, Fe, K, Mg, Na, Si, and Ti.

In order to investigate the influence of temperature and pressure on the characteristics of amorphous silica formation, the experiment was conducted with various combinations of temperature, pressure, and Si concentration adjusted for the supersaturation ratio of 2, as listed in Table 2. The influence of temperature was investigated with experiments at 430, 450, and 500 °C under the same pressure of 25 MPa, and the influence of pressure was investigated at 20, 25, and 30 MPa under the same temperature of 450 °C, where the residence time of water injected into the through-hole was estimated to be ≤ 10 s if assuming the density of pure water.

**Table 2:** Temperature, pressure, Si concentration, and residence time in the amorphous silica formation experiment at the expected supersaturation ratio of 2.

Temperature (°C)	Pressure (MPa)	Si concentration (mg kg^−1^)	Residence time (s)
430	25	98.2	7
450	25	98.2	6
500	25	98.2	5
450	20	49.4	4
450	30	193.3	8

### Amorphous silica transport experiment

In the amorphous silica transport experiment, pure water was first injected at 0.1 mL min^−1^ at 450 °C under a pressure of 30 MPa and confining stress of 40 MPa, and the injected fluid was then switched from pure water to Si-containing water with a prescribed Si concentration. The Si concentration was adjusted so that the supersaturation ratio of amorphous silica became either 2, 3, or 4 at the given combination of temperature and pressure, by diluting the Si-containing stock solution with pure water except in the case of the highest supersaturation ratio. As discussed later, nanoparticles were observed in all the amorphous silica formation experiments. Therefore, the temperature and pressure were not changed in this experiment. Effluent was collected after pure water within the flow path was completely replaced by Si-containing water. The effluent was filtered by the syringe filter to collect solids in the effluent for observation and analysis using SEM–EDX.

The amorphous silica transport experiment was conducted with various combinations of initial sample permeability and expected supersaturation ratio based on the adjusted Si concentration, as listed in Table 3, to investigate the influence of the initial permeability and amount of amorphous silica transported on the reduction of permeability in the fractured granite. The influence of initial permeability was investigated by conducting experiments using two samples having initial permeabilities of approximately 1 × 10^–17^ m^2^ and 1 × 10^–16^ m^2^ under the same supersaturation ratio of 4, and the influence of the amount of amorphous silica transported was investigated by conducting experiments at the supersaturation ratios of 2, 3, and 4 using samples with a similar initial permeability of approximately 1 × 10^–17^ m^2^.

**Table 3:** Initial permeability, Si concentration, and corresponding expected supersaturation ratio in the amorphous silica transport experiment conducted at 450 °C, 30 MPa, and a confining stress of 40 MPa.

Initial permeability (m^2^)	Si concentration (mg kg^−1^)	Expected supersaturation ratio
Approx. 1 × 10^–16^	380.6	4
Approx. 1 × 10^–17^	380.6	4
Approx. 1 × 10^–17^	290.0	3
Approx. 1 × 10^–17^	193.3	2

## Results and discussion

### Formation of amorphous silica nanoparticles

**Figure 2:**
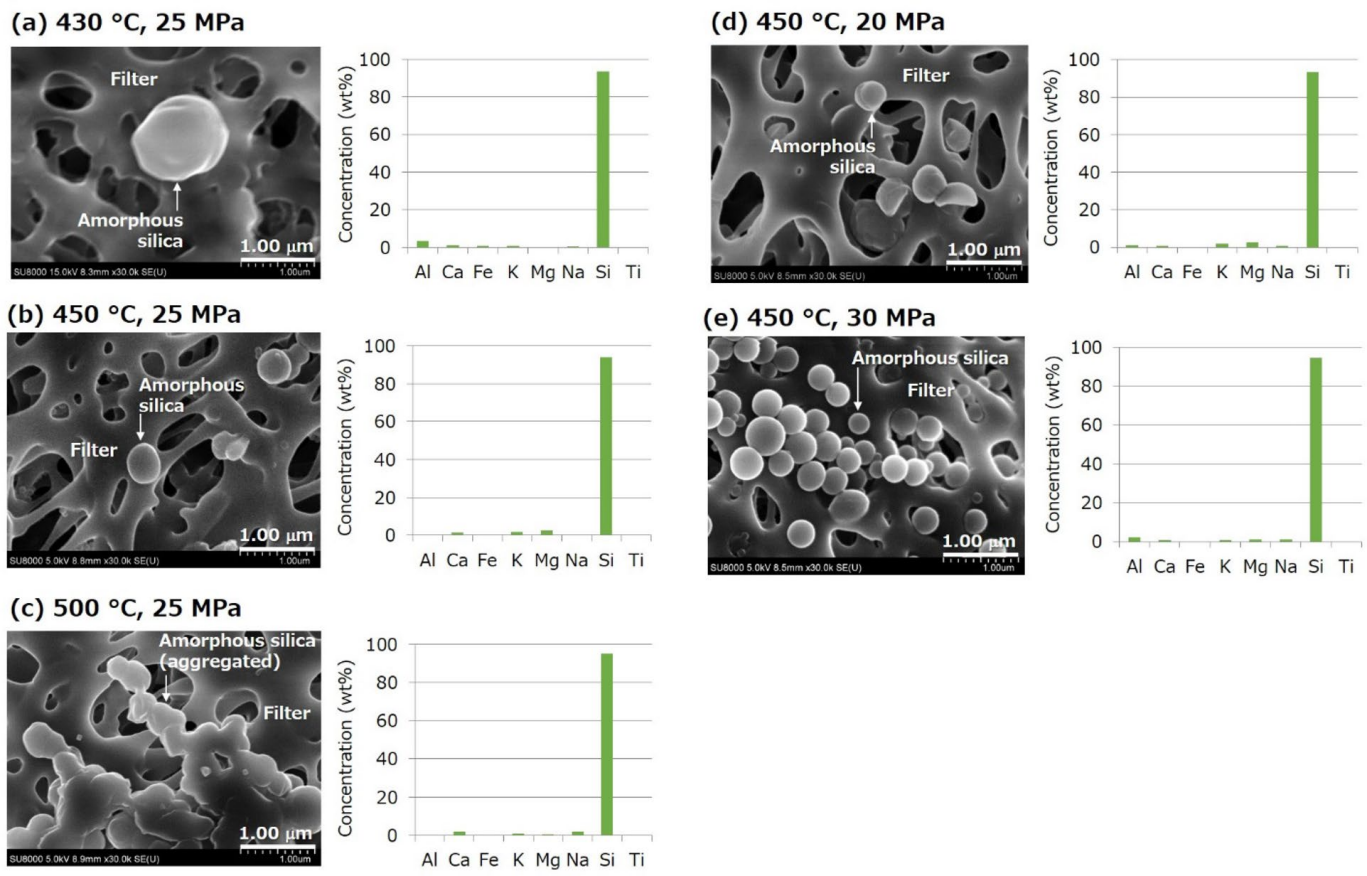
SEM images and elemental concentrations measured using EDX for nanoparticles formed in the amorphous silica formation experiments with various combinations of temperature and pressure: (**a**) 430 °C and 25 MPa, (**b**) 450 °C and 25 MPa, (**c**) 500 °C and 25 MPa, (**d**) 450 °C and 20 MPa, and (**e**) 450 °C and 30 MPa.

The formation of nanoparticles, which mainly contained Si, was identified using SEM–EDX for all the amorphous silica formation experiments (Fig. 2). It is well known that spherical silica particles having smooth surfaces are amorphous silica^36^. The characteristics of the formation of amorphous silica nanoparticles are dependent on temperature, where the particles become smaller with an increase in temperature and aggregate with each other at the highest temperature of 500 °C (Fig. 2a–c). The particle sizes were approximately 700–1200 nm (average 900 nm) at 430 °C, 200–500 nm (average 400 nm) at 450 °C, and 100–300 nm (average 200 nm) at 500 °C. On the other hand, pressure does not affect particle size significantly (Fig. 2b,d,e), and the particle size was 200–500 nm (average 400 nm) at all pressures. Note that the significantly large amount of amorphous silica nanoparticles at the highest pressure of 30 MPa may have been caused by a much higher Si concentration of injected water (Table 2) rather than the higher pressure.

**Figure 3:**
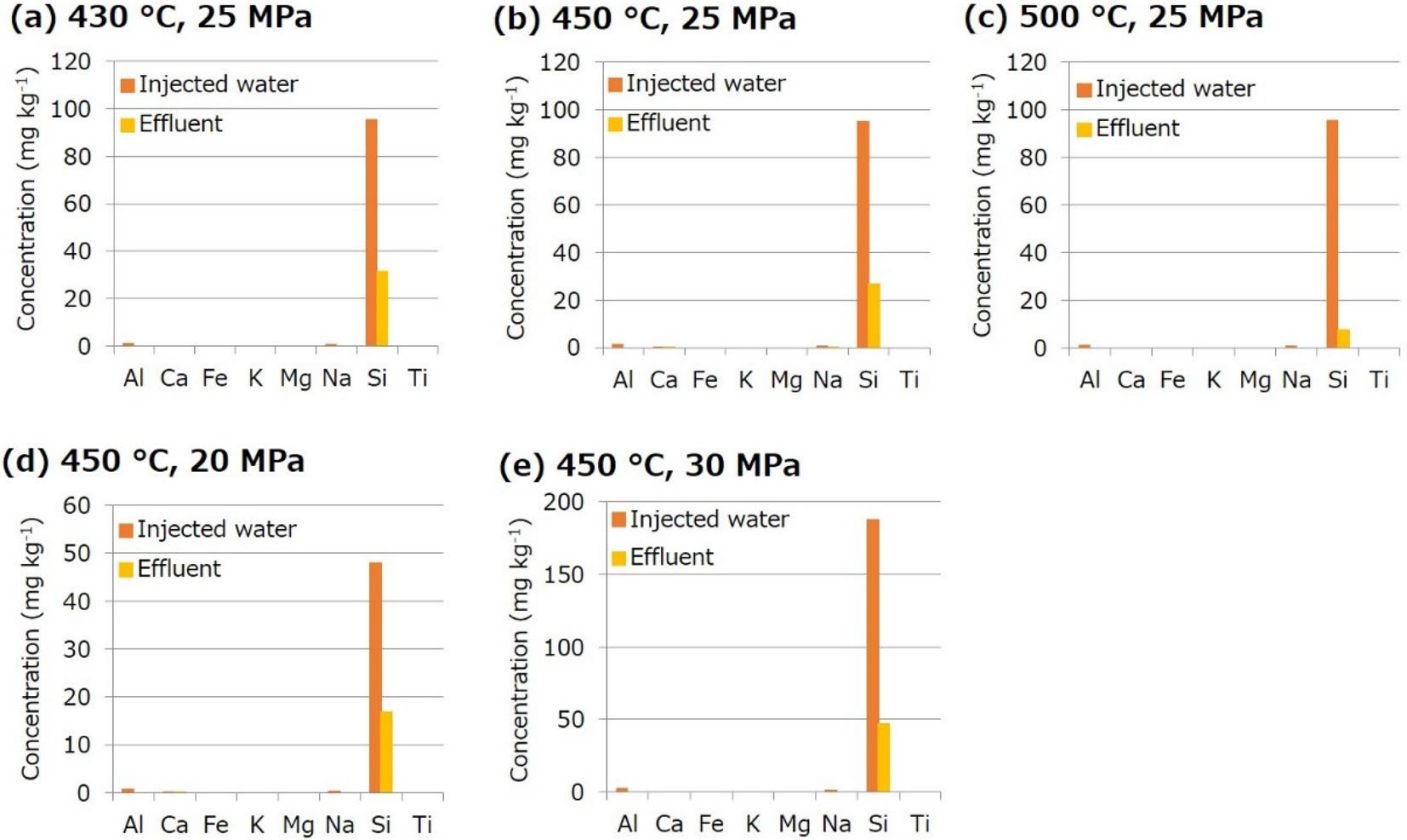
Elemental concentrations of the injected water and effluent in the amorphous silica formation experiments with various combinations of temperature and pressure: (**a**) 430 °C and 25 MPa, (**b**) 450 °C and 25 MPa, (**c**) 500 °C and 25 MPa, (**d**) 450 °C and 20 MPa, and (**e**) 450 °C and 30 MPa.

In all experiments, ICP–OES analysis revealed a large drop in the Si concentration of the effluent from that of the injected Si-containing water (Fig. 3). However, the Si concentration of the effluent was much smaller than the expected value (i.e., a half of the Si concentration of the injected water). This reveals the difficulty to accurately predict the amount of amorphous silica nanoparticles formed instantaneously by the phase change of water based on the solubility of amorphous silica due to non-equilibrium nature of the phenomenon and/or errors in the solubility prediction. In the present experiments, the expected supersaturation ratio was two for all conditions. However, it was approximately 3–12, where the difference between expected and actual supersaturation ratios tended to be larger for higher temperatures.

According to the classical theory of nucleation, the critical radius of the cluster *r*_*c*_, for homogeneous nucleation is as follows^40^:

1 $${r}_{c}=\frac{2\sigma v}{RTln\Omega },$$

where *σ* is the interfacial energy between the mineral and water, and *v* and Ω are the molar volume and the supersaturation ratio of the mineral, respectively, *R* is the universal gas constant, and *T* is the absolute temperature. This equation indicates that the nucleus size decreases with an increase in the temperature and supersaturation ratio, which is consistent with the present observations.

The stability of colloids in water at elevated temperature and pressure, including supercritical water, is primarily governed by the temperature dependence of the dielectric constant of water^41^. The interparticle electrostatic repulsive potential in the well-known DLVO theory^42,43^, which stabilizes the particles in the dispersion, decreases with an increase in the temperature because of the decrease in the dielectric constant of water^44^. For two identical spherical particles of radius *r*, with a Stern potential *ψ*_s_, and separated by a distance *H*, the repulsive potential *V*_*R*_ is expressed by

2 $$V_{R} = 2\pi \varepsilon \varepsilon_{0} r\psi_{s}^{2} ln\left\{ {1 + {\text{exp}}(-\kappa H)} \right\},$$

where *ε* is the dielectric constant of water, *ε*_0_ is the permittivity of the vacuum, and *κ* is the Debye–Hückel parameter. This equation indicates that the repulsive potential also decreases with the particle radius, which may explain the aggregation of the particles at the highest temperature.

### Reduction in the permeability of fractured granite

**Figure 4:**
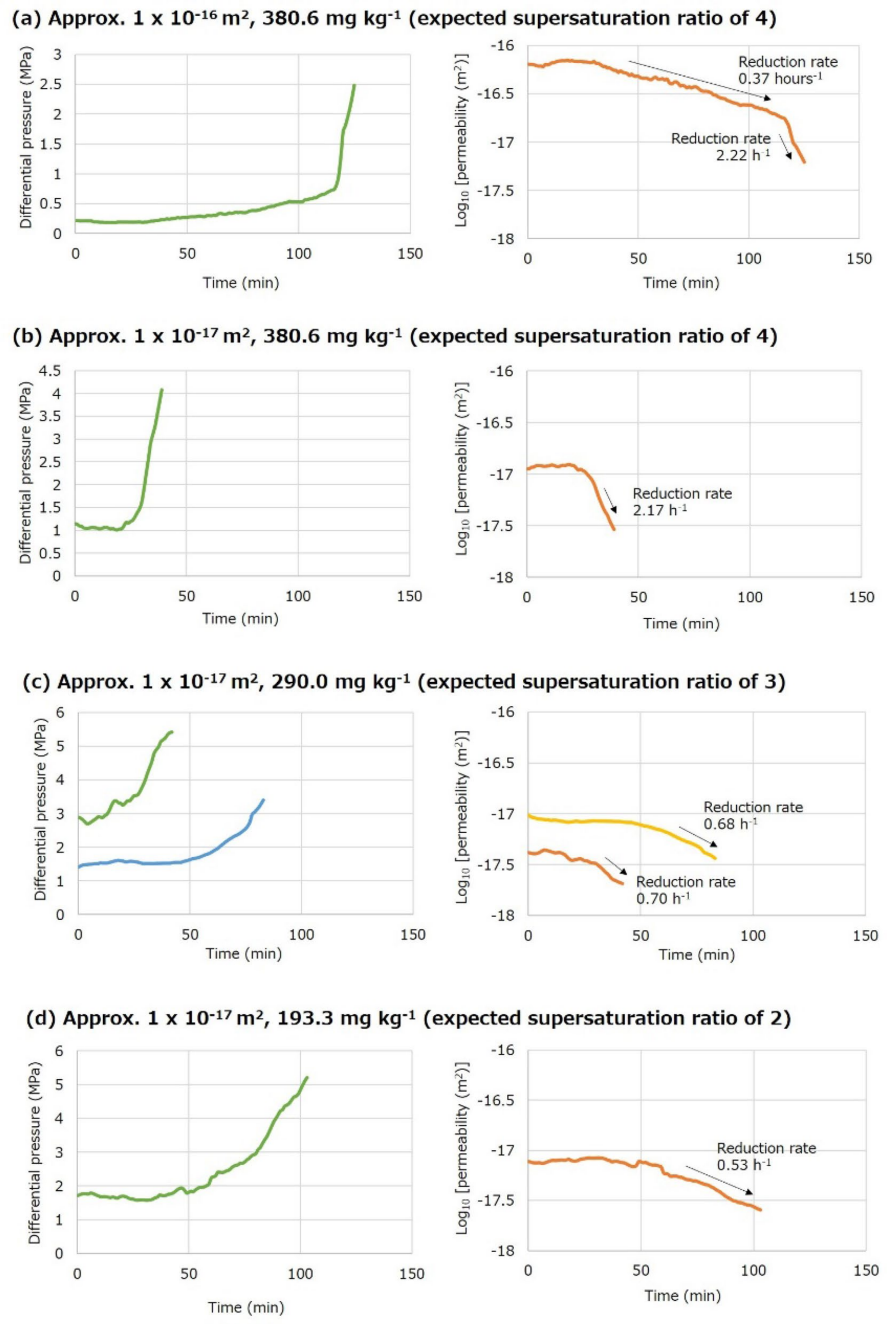
Changes in differential pressure and corresponding permeability in the amorphous silica transport experiments with various combinations of initial permeability and Si concentration (or corresponding expected supersaturation ratio): (**a**) approximately 1 × 10^–16^ m^2^ and 380.6 mg kg^−1^ (expected supersaturation ratio of 4), (**b**) approximately 1 × 10^–17^ m^2^ and 380.6 mg kg^−1^ (expected supersaturation ratio of 4), (**c**) approximately 1 × 10^–17^ m^2^ and 290.0 mg kg^−1^ (expected supersaturation ratio of 3), and (**d**) approximately 1 × 10^–17^ m^2^ and 193.3 mg kg^−1^ (expected supersaturation ratio of 2).

**Figure 5:**
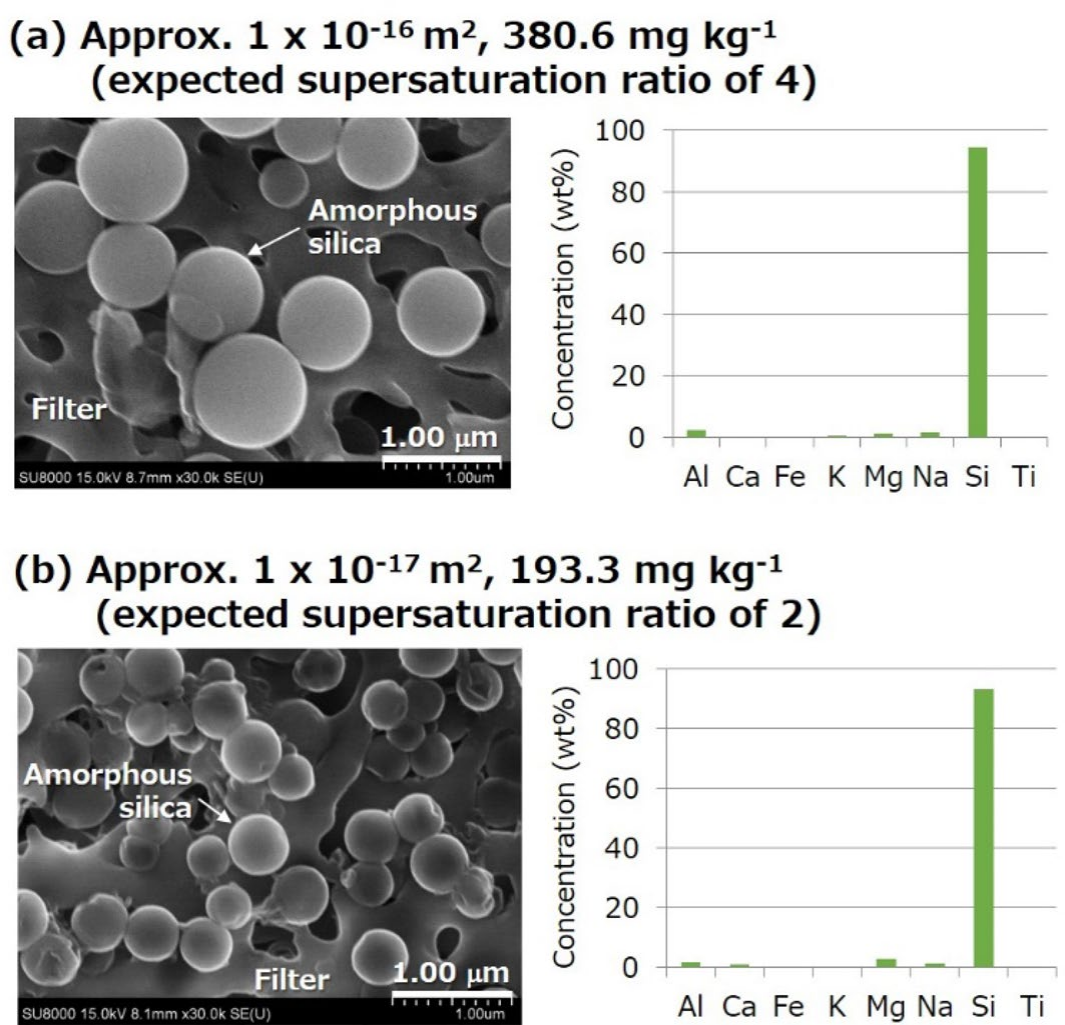
SEM images and elemental concentrations measured using EDX for nanoparticles that passed through the fractured granite in the amorphous silica transport experiments with different combinations of initial permeability and Si concentration (or corresponding expected supersaturation ratio): (**a**) approximately 1 × 10^–16^ m^2^ and 380.6 mg kg^−1^ (expected supersaturation ratio of 4), and (**b**) approximately 1 × 10^–17^ m^2^ and 193.3 mg kg^−1^ (expected supersaturation ratio of 2).

A rapid and significant decrease in sample permeability was observed within several hours in all the amorphous silica transport experiments (Fig. 4); the experiment was conducted twice at the smaller initial permeability of approximately 1 × 10^–17^ m^2^ and the expected supersaturation ratio of 3 to check reproducibility. The presence of amorphous silica nanoparticles that passed through the fractured granite sample was confirmed by SEM–EDX for two experiments (Fig. 5), in which the injection of Si-containing water lasted for a relatively longer duration to collect sufficient effluent (Fig. 4a,d).The particle size was approximately 500–1000 nm (average 800 nm) at the initial permeability of approximately 1 × 10^–16^ m^2^ and the expected supersaturation ratio of 4 (Fig. 5a), and 200–500 nm (average 400 nm) at the initial permeability of approximately 1 × 10^–17^ m^2^ and the expected supersaturation ratio of 2 (Fig. 5b).

In all experiments, the differential pressure and corresponding permeability, which were approximately constant in the early stage of the experiment, were respectively increased and decreased with time after Si-containing water reached the inlet side of the sample (Fig. 4). In the experiment with a higher initial permeability of approximately 1 × 10^–16^ m^2^ (Fig. 4a), the permeability reduction occurred in two stages, where the rate of permeability reduction increased significantly after the permeability became close to 1 × 10^–17^ m^2^. Regardless of the number of stages, the reduction in the logarithmic permeability (log_10_ [permeability (m^2^)]) was approximately linear in each stage for all experiments, which allows for the determination of the rate of permeability reduction (in h^−1^) as the absolute value of the slope of the linear regression curve by the least squares method. The smaller and larger rates of permeability reduction in the first and second stages were 0.37 h^−1^ and 2.22 h^−1^, respectively (Fig. 4a), where the rate in the second stage was similar to that with the smaller initial permeability of approximately 1 × 10^–17^ m^2^, namely 2.17 h^−1^ (Fig. 4b). Therefore, the rate of permeability reduction due to clogging by amorphous silica particles was smaller for fractured granite with higher permeability, which may be due to the larger aperture of the fractures.

**Figure 6:**
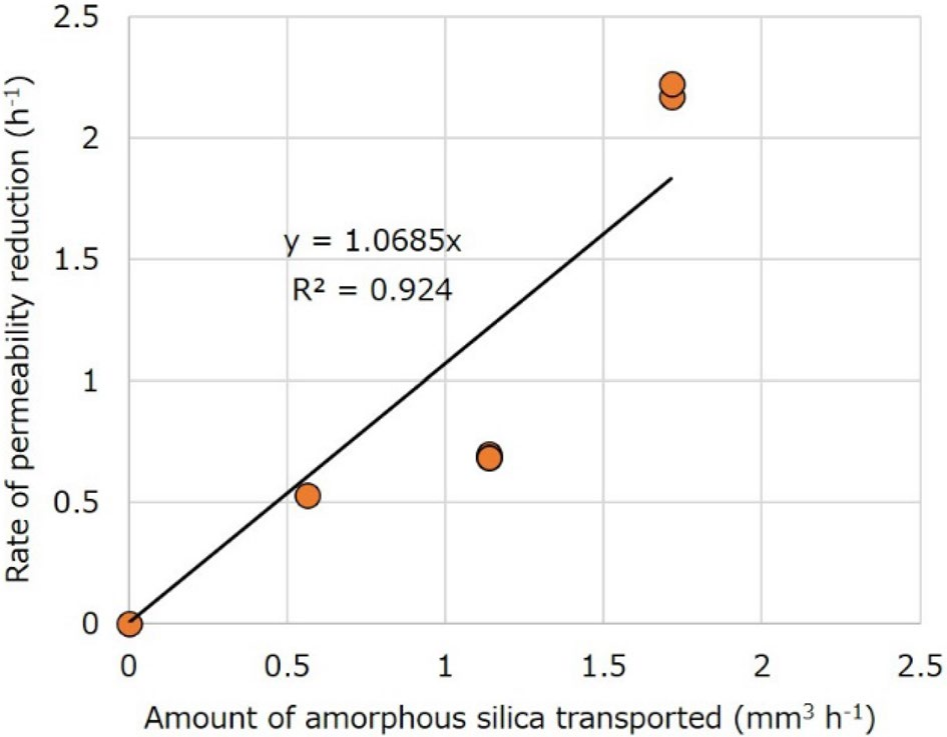
Relation between the rate of permeability reduction and the amount of amorphous silica transported in the amorphous silica transport experiment.

On the other hand, at similar initial permeabilities of approximately 1 × 10^–17^ m^2^, the rate of permeability reduction decreased with a reduction in the expected supersaturation ratio and thus the Si concentration of injected water. The permeability reduction rates at supersaturation ratios of 4, 3, and 2 were respectively 2.22 h^−1^ (the second stage in Fig. 4a) and 2.17 h^−1^ (Fig. 4b), 0.68 and 0.70 h^−1^ (Fig. 4c), and 0.53 h^−1^ (Fig. 4d). This implies that the permeability reduction rate is a function of the amount of amorphous silica transported per hour, which may be approximately estimated from the flow rate (0.1 mL min^−1^), density of amorphous silica (2.2 × 10^3^ kg m^−3^), and the drop in the Si concentration expected from the concentration of the injected water and the solubility of amorphous silica during nanoparticle formation^38,39^, although it may not be identical to (may be smaller than) the actual amount. The relation between the permeability reduction rate and the amount of amorphous silica transported is positive, and approximately linear if a zero rate is assumed for no amorphous silica formation (Fig. 6).

The amount of amorphous silica transported was estimated to be less than 2 mm^3^ h^−1^, as shown in Fig. 6. On the other hand, the pore volume of the fractured granite with a permeability of 1 × 10^–17^ m^2^ was estimated to be approximately 111 mm^3^, which corresponded to the fractured granite sample of approximately 17,671 mm^3^ having fractional porosity of approximately 0.0063, based on the following empirical relation between the fractional porosity (*ϕ*) and the permeability (*k* in m^2^) reported for the same type of granite containing thermally induced multiple fractures^26^:

3 $${\mathrm{log}}_{10}\,\phi = 0.19\,{\mathrm{log}}_{10}\,k+1.03.$$

Consequently, the reduction of pore volume or porosity per hour due to the deposition of amorphous silica was negligibly small. Moreover, even if the amount of amorphous silica transported was larger than expected by an order of magnitude, for instance 20 mm^3^ h^−1^, then the rate of permeability reduction due to the reduction of porosity would be only 0.45 h^−1^ based on Eq. (3), which is not consistent with the observation. These results imply that the amorphous silica nanoparticles clog relatively small apertures along dominant flow paths in fractured granite during their transport by fluid flow.

### Risk of permeability reduction in superhot EGS

The present study has demonstrated that amorphous silica occurs in the form of nanoparticles in both supercritical water and superheated steam at various combinations of temperature and pressure in superhot geothermal environments when a supersaturated state is induced by the phase change of Si-containing subcritical water. It has also been revealed that the nanoparticles can reduce the permeability of the fractured granite significantly and rapidly by clogging the fractures. The permeability reduction rate due to clogging, as observed in the present study, is quite fast when compared with previously reported rates of permeability reduction due to water–rock reactions for fractured and intact granite under confining stress, ≤ 10^–1^ days at temperatures up to 500 °C^22–24^.

In a superhot EGS, amorphous silica nanoparticles may occur between injection and production wells in which the phase change inevitably occurs with an increase in the temperature of the injected water, and such nanoparticles may reduce the permeability of fractured rock by clogging the fractures and finally transforming to quartz, as suggested by Amagai et al.^36^. The present study thus supports the suggestion in Amagai et al.^36^ that such short-lived silica particles can have a significant impact on the dynamic changes in the mechanical behavior and hydrology of hydrothermal systems in volcanic areas. Based on the present study, for instance, an impermeable zone is likely to form at a depth where the phase change from subcritical water to supercritical water occurs, as suggested in Saishu et al.^20^.

However, as observed in the present study, the amorphous silica nanoparticles formed in superhot geothermal environments may pass through fractured rocks to be finally produced from the production well, when the fractures created in superhot EGS by hydraulic fracturing^34,35^ have much larger apertures than the nanoparticles. Moreover, as observed in the present study, the rate of permeability reduction of a fractured rock may be decreased with a reduction in the amount of amorphous silica transported, even if all of nanoparticles cannot pass through the fractures. Consequently, the risk of permeability reduction due to the formation of amorphous silica nanoparticles may be decreased by an increase in large-aperture fractures and a reduction in the amount of amorphous silica transported through them.

For instance, large-aperture fractures may be increased as a result of enhanced free-face dissolution in a suppressed pressure solution for fracture surfaces during high-pressure water injection in hydraulic fracturing^24^. It has been suggested that an increase of the fracture aperture size in granite can occur in superhot geothermal environments when the rate of free-face dissolution (increase of aperture size) becomes faster than the rate of the pressure solution (decrease of aperture size). With increasing pressure, free-face dissolution becomes faster due to an increase in mineral solubility and, in contrast, the pressure solution becomes slower due to the decrease in the effective confining stress. Thus, high-pressure water injection may contribute to an increase in large-aperture fractures.

On the other hand, the amount of amorphous silica transported may be reduced by inducing a phase change from subcritical water to supercritical water at relatively high pressures. Cold water injected from an injection well will dissolve rocks with an increase in the temperature and the Si concentration will then reach a maximum around the critical temperature of water. The maximum Si concentration would be, for instance, approximately 350 mg kg^−1^ at 20 MPa, 400 mg kg^−1^ at 30 MPa, and 480 mg kg^−1^ at 40 MPa, based on the solubility of quartz^39^. The subsequent temperature rise induces the phase change of the injected water to superheated steam or supercritical water, which produces amorphous silica nanoparticles of which the amount will depend on the drop in the Si concentration (i.e., the maximum Si concentration minus the Si concentration estimated from the solubility of amorphous silica after the phase change). At 400–500 °C, for instance, the Si concentration based on the solubility of amorphous silica would be approximately 30 mg kg^−1^ at 20 MPa, 80–500 mg kg^−1^ at 30 MPa, and 210–930 mg kg^−1^ at 40 MPa, where the concentrations at 30 MPa and 40 MPa are higher for higher temperatures^38,39^. Therefore, the drop in the Si concentration is approximately 320 mg kg^−1^ at 20 MPa, 0–320 mg kg^−1^ at 30 MPa, and 0–270 mg kg^−1^ at 40 MPa, which suggests a lower risk of permeability reduction for a higher-pressure circulation of water between injection and production wells during geothermal energy production.

## Conclusions

In the development of enhanced geothermal systems using superhot EGS, the formation of amorphous silica nanoparticles may inevitably occur, with smaller particles for higher temperature, between injection and production wells due to a phase change from subcritical water to supercritical water or superheated steam. The nanoparticles may be transported through fractured rocks to finally clog or pass through the fractures, depending on the size of the fracture apertures. With smaller aperture fractures, the permeability of fractured rocks may decrease rapidly and significantly, with higher rates at larger numbers of transported nanoparticles. Consequently, the risk of permeability reduction due to the formation of amorphous silica nanoparticles should be considered and reduced as much as possible in the development of superhot EGS. Some possible methods to reduce the risk are to increase larger-aperture fractures using the free-face dissolution of fracture surfaces in forming reservoirs, and avoiding phase change at lower pressures during the extraction of thermal energy from the reservoirs. Further detailed studies are encouraged to investigate the risk of permeability reduction and to develop countermeasures to reduce permeability reduction and achieve sustainable and profitable extraction of superhot geothermal energy.

## Data Availability

The data that support the findings of this study are available from the corresponding author on reasonable request.
